# Pulling strength, muscular fatigue, and prediction of maximum endurance time for simulated pulling tasks

**DOI:** 10.1371/journal.pone.0207283

**Published:** 2018-11-16

**Authors:** Cannan Yi, Kai Way Li, Fan Tang, Huali Zuo, Liang Ma, Hong Hu

**Affiliations:** 1 School of Safety & Environmental Engineering, Hunan Institute of Technology, Hengyang City, Hunan Province, China; 2 Department of Industrial Management, Chung Hua University, Hsin-Chu, Taiwan; 3 Department of Industrial Engineering, Tsinghua University, Beijing, China; University of Essex, UNITED KINGDOM

## Abstract

Truck pulling is one of the common manual materials handling tasks which contribute to musculoskeletal disorders. The maximum endurance time (MET) for two-handed truck pulling tasks has been rarely discussed in the literature. The objectives of this study were to explore the development of muscular fatigue when performing two-handed pulling task and to establish models to predict the MET. A simulated pallet truck pulling experiment was conducted. Sixteen healthy adults including eight females and eight males participated. The participants pulled a handle simulating that of a pallet truck using two hands until they could not pull any longer under two postures. The forces applied for females and males were 139.65 N and 170.03 N, respectively. The maximum voluntary contractions (MVC) of the pulling strength both before and after the simulated pull were measured. After each trial, both the MET and subjective ratings of muscular fatigue on body segments were recorded. The results showed that posture significantly affected MVC of pull both before and after the trial. It was found that foot/shank of the front leg had higher subjective ratings of muscular fatigue than the other body segments. The MET equations employing both power and logarithmic functions were developed to predict the MET of the two-handed pulling tasks. Predictive models established in this study may be used to assess the MET for two-handed pulling tasks.

## Introduction

Manual Material handlings (MMH) are common at workplaces. They contribute to the occurrence of work-related musculoskeletal disorders (MSDs) [1]. Carts, trolleys, and pallet trucks are commonly used materials handling aids [2]. These aids are either pushed or pulled manually. A survey [3] conducted in automotive supply sectors showed that approximately 10% of all working processes involved pushing or pulling. Forty one percent of the materials handled in one transport were between 200 kg and 1000 kg. In the USA, 20% of all industrial back injuries were associated with pulling or pushing tasks [4]. Repetitive force exertion, overloading, long time exposure, and unnatural posture when performing the MMH tasks have been recognized as the main causes of the MSDs [5].

Manual-operated pallet trucks are widely used for materials handling [6]. Mack et al. [7] found that 40% of the workers in the material handling sector they visited used a truck more than 10 times per day. St-Vincent et al. [8] has shown that the pallet trucks in the warehouse they visited were used 53 times, on average, in a five-hour work shift and were used as many as 93 times in some sectors. It was found that the cumulative mass handled by a pallet truck in a day ranged from approximately 300 to 4400 kg [8]. According to the Bureau of Labor Statistics [9], a total of 2,710 lost-time injuries occurred because of pallet truck use in the USA. Workers operating manual-operated pallet truck were suffering high risk of MSDs, especially on their back and upper extremities [10]. To reduce the risk of MSDs, many studies have been performed to explore the effects of heights, loads, velocities, angles of pulling on the development of muscular fatigue [11, 12]. Design and redesign of the materials handling devices to lower pulling burden have also been proposed [6, 10, 13].

There are many symptoms associated with MSDs. Frequent muscular fatigue is one of them. Muscular fatigue can be defined as “reduction in the ability to exert force in response to voluntary effort” [14]. It can be quantified by assessing the reductions of maximum voluntary contraction (MVC) before and after the forceful exertion [15, 16] and the changes of the electromyography (EMG) of muscles upon forceful exertions [17, 18]. Muscular fatigue may also be assessed subjectively. The ratings of percei**v**ed exertion have been widely used for such assessments [19].

The maximum endurance time (MET) is also associated with muscular fatigue and has been adopted in ergonomic guidelines [20]. It represents the maximum time during which a static muscular load can be maintained [21]. Alternatively, the MET may be defined as the maximum time that a worker could perform a physical task under a specific force exertion condition. The MET data for specific tasks may be used to determine the time period that workers may be able to work without a pause due to muscular fatigue. MET models have been proposed to estimate the MET for workers performing physical tasks when the real MET data are not available in industrial settings [20]. Both theoretical and empirical MET models have been reported [22]. The theoretical MET models were established by developing mathematical equations to represent the developing of muscular fatigue [14]. The empirical MET models, on the other hand, were developed by fitting the experimental data to mathematic functions under specific body segment and forceful exertion conditions.

A pallet truck may be pulled using either one hand or two hands. Two-handed pulling is adopted for heavy materials handling. It, as compared with the one-handed pulling, is associated with more forceful exertions which could result in more muscular fatigue problems. Although the literature has discussed muscular fatigue for pulling tasks [6, 10, 13, 23–25], they were mainly focused on the decrease of muscular strength. The significance of MET data and the establishment of MET models for one-handed tasks have been discussed in two of our articles [26, 27]. Whether the existing MET models [20, 26–34] in the literature are applicable to the two-handed pulling tasks are questionable as none of them were developed specifically under two-handed pulling conditions.

The objective of this study was to investigate the developing of muscular fatigue for static two-handed truck pulling tasks via measuring the pulling strength both before and after the pull. The METs and subjective ratings of muscular fatigue were examined. In addition, predictive models were established as tools to predict the MET for two-handled pulling tasks. These models are significant as they may be used to predict the MET so as to determine the work-rest allowance for workers where two-handed pulling tasks are performed.

## Methods

A simulated pallet truck pulling experiment was conducted in the laboratory of the Hunan Institute of Technology in China. This study was approved by an Ethical Review Committee of the Institute. The temperature and humidity during the experiment were 17.35°C (SD = 4.02) and 87.45% (SD = 11.68), respectively.

### Participants

A call for participations in the experiment was announced in the campus where the authors served. Any adult without self-reported MSDs problems within a year of the study was welcomed. Sixteen participants (8 males and 8 females) joined voluntarily. All of them were right-handed. The research personnel explained the purposes and procedure of the experiment to the participants at their first appearance in the laboratory. All the participants read and signed informed consent before participating in the experiment. They wore their own sport shoes in the study. Fundamental anthropometric data of these participants are shown in Table 1.

**Table 1:** Anthropometrics of the participants.

Variables	Female	Male
Age (years)	20.50(1.68)	22.13(1.56)
Weight (kg)	51.46(2.50)	61.00(7.18)
Stature (cm)	162.63(1.54)	163.71(1.92)
BMI (kg/m^2^)	19.46(0.88)	22.74(2.47)
Arm length (cm)	61.63(1.26)	63.41(2.31)
Leg length (cm)	88.88(3.74)	92.45(2.58)
Knee height (cm)	47.19(2.08)	47.15(3.96)
Shoulder height (cm)	134.31(1.39)	135.54(2.06)

Note: Values within brackets are standard deviations.

### Apparatus

**Fig 1:**
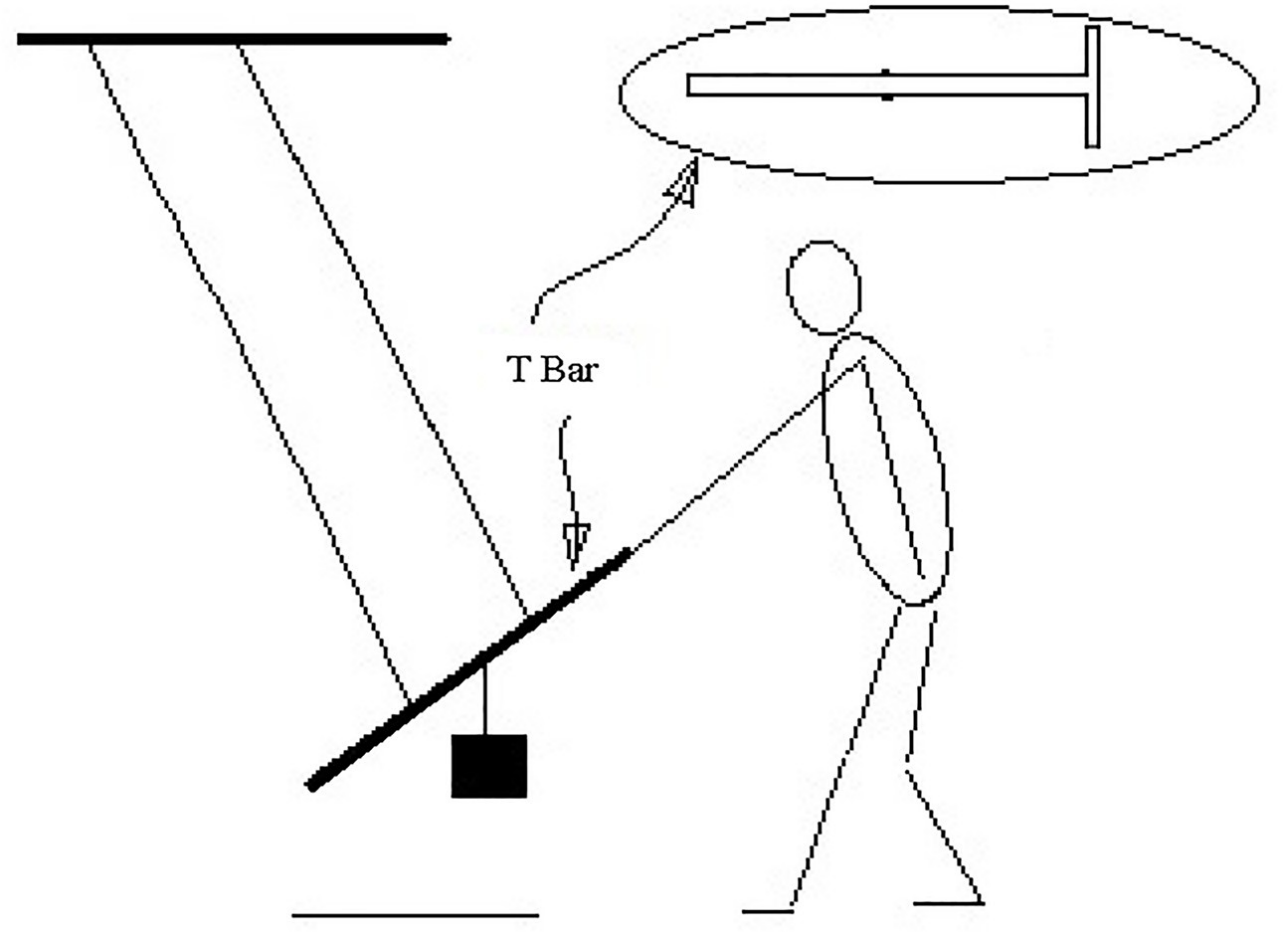
Suspended T bar and the simulated pulling task.

A simulated pulling experiment was designed. The authors have fabricated a steel T-bar mimic the stick and handle of a real pallet truck available in the market [26, 27]. This bar has a weight of 1.5 kg with a length of 81.5 cm. One side of this bar has a handle with a diameter of 3.0 cm. Two wires were adopted to suspend this bar from the ceiling. A weight was suspended in the middle of the bar to generate a back swing force (see Fig 1).

The weight was 40 kg and 50 kg for female and male participants, respectively. The forces required to counterbalance the back swing forces for these two loads were 139.65 N and 170.03 N, respectively. The reason to select these two loads was that we wanted to control each of the experimental trial lasting no more than 15 min. In practice, a one-time handling of materials lasting more than 15 min is preferably to be handled using an powered pallet jack which is not within the scope of our study. The literature [1] indicated that workers could work for an eight hour shift without excess muscular fatigue at the end of the day when the physiological work load was no more than a third of the MVC. For the 40 kg and 50 kg external loads, the %MVC of the pulling force for male and female participants were between 43% and 68% and between 47% and 78%, respectively.

**Fig 2:**
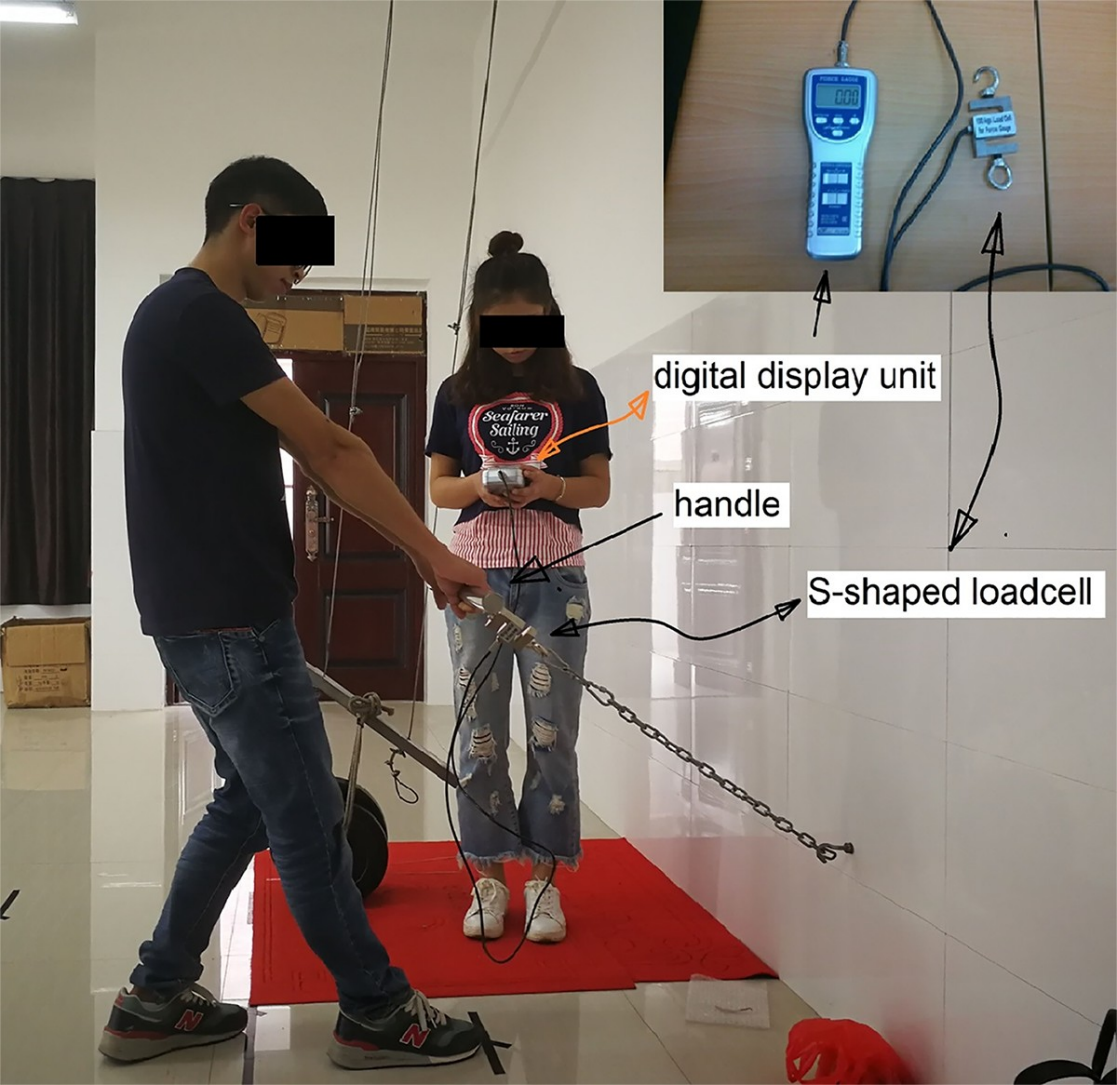
Pulling strength testing. **Note:** The individual in this photo has given written informed consent (as outlined in PLOS consent form) to publish these case details.

The participants pulled the handle statically during the trials using two hands. The pulling strength was measured using a strength measuring apparatus. This apparatus included a chain connected to a hook 37 cm above the ground on the wall, an S-shaped loadcell (Lutron^®^ Inc., FG-5100), and a handle 3 cm in diameter. When the participant pulled the handle (see Fig 2), a digital display showed the peak force of the pulling.

A stopwatch was utilized to measure the MET. A Borg CR-10 rating scale was employed to record the levels of muscular fatigue (0 = no fatigue at all to 10 = extremely fatigue) in different body segments of the participant after each pulling trial. This scale is easy to administer and has been adopted in the literature [15, 26–28, 32] concerning muscular fatigue.

### Procedure

For each participant, a practice was carried out before the experiment. The participant pulled a real truck without external load to get familiar with the posture of truck pulling. The participant adopted and maintained this posture in the following simulated truck pulling tasks.

Before each trial, the participant was required to do a warm-up exercise, following an aerobic fitness video, for 5 minutes. Then, the maximum pulling strengths of the participant was measured. In this measurement, the participant was instructed to pull the handle using their hands as hard as he or she could for 4 to 6s without jerking (see Fig 2). The reading of this measurement was recorded as MVC_before._

After the strength measurement, the participant put the handle down on the floor and jointed a simulated truck pulling task. In this task, the participant pulled the T bar duplicating the posture in the real truck pulling conditions (see Fig 1) until he or she could no longer do so. The maximum time they could pull was recorded as the MET. There were two postures in the pulling. The pulling with left foot in the front was termed posture 1 while posture 2 was with the right foot in the front. Repeated trials under each posture were tested. These comprised four different trials. The order of the trial was selected randomly. After each trial, the maximum pulling strength of the participant was measured again and were denoted as MVC_after_. The CR-10 of the left hand (LH), left shoulder (LS), right hand (RH), right shoulder (RS), waist (WT), left foot/shank (LF) and right foot/shank (RF) were recorded. After completing one trial, the participant was dismissed and was requested to return for the next trial the next day or after. There was a pause of at least 12 hours between any two trials. The participants were instructed not to take strenuous exercise or activities at least four hours before they came to the laboratory for the experiment.

### Data processing

A total of 64 pulling strengths and METs (2 postures×2 repetitions×2 genders × 8 participants) and 448 CR-10 scores (2 postures×2 repetitions×2 genders ×8 participants×7 body segments) were measured to explore the developing of muscular fatigue in simulated pallet truck pulling task. Test of normality for the pulling strength data was performed using the Kolomogorov-Smirnov test. Descriptive statistical analysis was conducted. Pearson correaltion coefficients among MVC_before_, MVC_after_, MET and CR-10 scores were calculated. To determine the effects of posture and gender on MVC_before_, MVC_after_, and MET, analyses of variance (ANOVA) were carried out. The rank-based Kruskal-Wallis tests were performed to test the effects of posture on the CR-10 score. Regression analyses were conducted to establish the MET models. These models were verified by comparing measured data, predicted data of our models and that of the models in the literature. The data collected was processed using Excel 2010. SAS 9.4 software were adopted for statistical analyses. Statistical significance was set at *p*<0.05.

### MET modeling

MET is normally calculated using the %MVC or relative force (f_*MVC*_ = %MVC/100) required by the task[21]. MET- %MVC or MET- f_*MVC*_ relationship has been long recognized to be negative nonlinear and has been fitted using three mathematic functions [14, 21]: power, exponential and logarithmic. We, then, adopted these four functions to fit our MET equation for truck pallet pulling tasks:

$$MET=a\times e^{f_{MVC}^{}\times b}$$ (1)

$$MET=a\times f_{MVC}^{b}$$ (2)

$$MET=a\times \ln(f_{MVC})$$ (3)

For Eqs (1) and (2), Eqs (4) and (5) were obtained via logarithm transformation:

$$\ln(MET)=\ln(a)+b\times f_{MVC}$$ (4)

$$\ln(MET)=\ln(a)+b\times \ln(f_{MVC})$$ (5)

For Eq (3), let *x* = ln(*f*_*MVC*_), we have

MET=a×x (6)

Eqs (1) to (3) may be fitted using simple linear regression analysis if the MET and f_*MVC*_ are known. To compare the difference between the measured and predicted MET, a mean absolute deviation (MAD) was often used [15, 35],

$$\mathrm{MAD}=\frac{1}{n}\overset{n}{\underset{i=1}{\sum }}|\mathrm{measured}\;\mathrm{value}–\mathrm{predicted}\;\mathrm{value}|$$ (7)

## Results

### Pulling strength

The Kolomogorov-Smirnov test results confirmed the normality of the pulling strength data (*p*>0.15). Table 2 shows the means and standard deviations of the MVC_before_, MVC_after_, and MET over gender and postures conditions. We assumed that posture significantly affected muscular fatigue progressing. The results, however, showed that posture only affected the MVC_before_ (F(1,60) = 4.58, *p*<0.05) and MVC_after_ (F(1,60) = 5.2, *p*<0.05) significantly. The MVC_before_ and MVC_after_ for all participants of posture 1 (284.35 N and 217.67 N, respectively) were significant lower than those of posture 2 (298.75 N and 232.05 N, respectively). Normally, right-hander involuntarily put their right foot on the front when they pulled. They could exert more force with their right foot on the front than that of left foot on the front. This might contribute to the difference for both the MVC_before_ and MVC_after_ between posture 1 and posture 2. A comparison of the MVC_before_ between genders was conducted. The MVC_before_ of males (304.02 N) was significant higher (F(1,60) = 13.73, *p<*0.001) than those of females (279.09 N). The MVC_before_ of females was 92% to that of their male counterparts. Person correlation results showed that posture was insignificant to the MET (*p*>0.05).

**Table 2:** MVC_before_, MVC_after_ and MET over gender and postures.

	Female		Male	
	Posture 1	Posture 2	Posture 1	Posture 2
MVC_before_(N)^*^^†^	274.22 (15.51)	283.96 (33.64)	294.49 (31.45)	313.54 (23.13)
MVC_after_(N)^**†**^	201.91 (17.05)	224.68 (34.44)	233.42 (25.07)	239.43 (21.01)
MET(min)	11.06 (2.49)	10.97 (3.48)	9.85 (2.43)	9.43 (2.24)

Note: Values within brackets are standard deviations.

^*^significant at *p*<0.05 for gender

^**†**^significant at *p*<0.001 for posture.

### CR-10 ratings

Posture was insignificant to overall CR-10 rating. The effects of posture on the CR-10 rating for each body segment were tested. Posture was found to be significant to the CR-10 ratings for both LF (χ_1_^2^ = 39.80, *p*<0.0001) and RF (χ_1_^2^ = 31.97, *p*<0.0001). The CR-10 ratings on the leading foot were significantly higher than that of the lag foot. The effects of posture on the CR-10 rating on all other body segments were not significant. Table 3 shows the CR-10 scores for each body segment. For both postures, the leading foot had the highest scores, followed by the hands, waist and other body segments. Difference between the two hands was not significant. Person correlation results showed that the CR-10 scores of some body segments (WT, LF and RF) were insignificant to the MET (*p*>0.05) while the CR-10 of the other body segments (LH, LS, RH and RS) were significant with a low correlation coefficient (*r*< |0.5|, *p*<0.05) to the MET.

**Table 3:** CR-10 ratings on body segments.

Posture 1			Posture 2		
Body segments*	Mean	(SD)	Body segments**	Mean	(SD)
LF	7.22	(1.41)	RF	6.63	(1.60)
LH	5.56	(1.37)	RH	5.81	(1.65)
RH	5.38	(1.72)	LH	5.00	(1.95)
WT	4.53	(1.50)	WT	4.41	(1.83)
RS	4.06	(1.61)	RS	3.97	(1.75)
LS	3.88	(1.41)	LF	3.56	(1.52)
RF	3.72	(1.42)	LS	3.41	(1.96)

*χ_6_^2^ = 79.72, *p*<0.0001

**χ_6_^2^ = 65.86, *p*<0.0001.

### MET results

The MET for male and female participants were 11.02 min (SD = 2.98) and 9.61 min (SD = 2.31), respectively. The difference between the two genders was not statistically significant. The MET differences between repeated trials ranged from 0.067 to 4.9 min, with average value of 2.14 (SD = 1.28) min and average coefficient of variation (CV) (%) of 13.27 (SD = 8.15). The CV of the MET for each participant between two trials ranged from 0.59% to 31.07%, with an average of 14.91% (SD = 8.61). The Shapiro-Wilk test result supported the normality of the MET (W = 0.986, *p* = 0.70). Regression analyses were performed for Eqs (4) to (6). The MET equations in Table 4 could be used to predict endurance time for female and male respectively when performing two-handed manual pallet truck pulling tasks.

**Table 4:** MET predictive models.

Regression equation		*R*^*2*^	*p-value*	MAD (min)
Female				
$MET=f_{MVC}^{-3.39331}$	(8)	0.98	*<0*.*0001*	2.62 (1.82)
*MET* = −15.96043ln(*f*_*MVC*_)	(9)	0.95	*<0*.*0001*	2.14 (1.59)
Male				
$MET=f_{MVC}^{-3.80906}$	(10)	0.98	*<0*.*0001*	2.26 (1.53)
*MET* = −16.5854ln(*f*_*MVC*_)	(11)	0.96	*<0*.*0001*	1.61 (1.17)

Note: Values within brackets in the MAD column are standard deviations.

**Fig 3:**
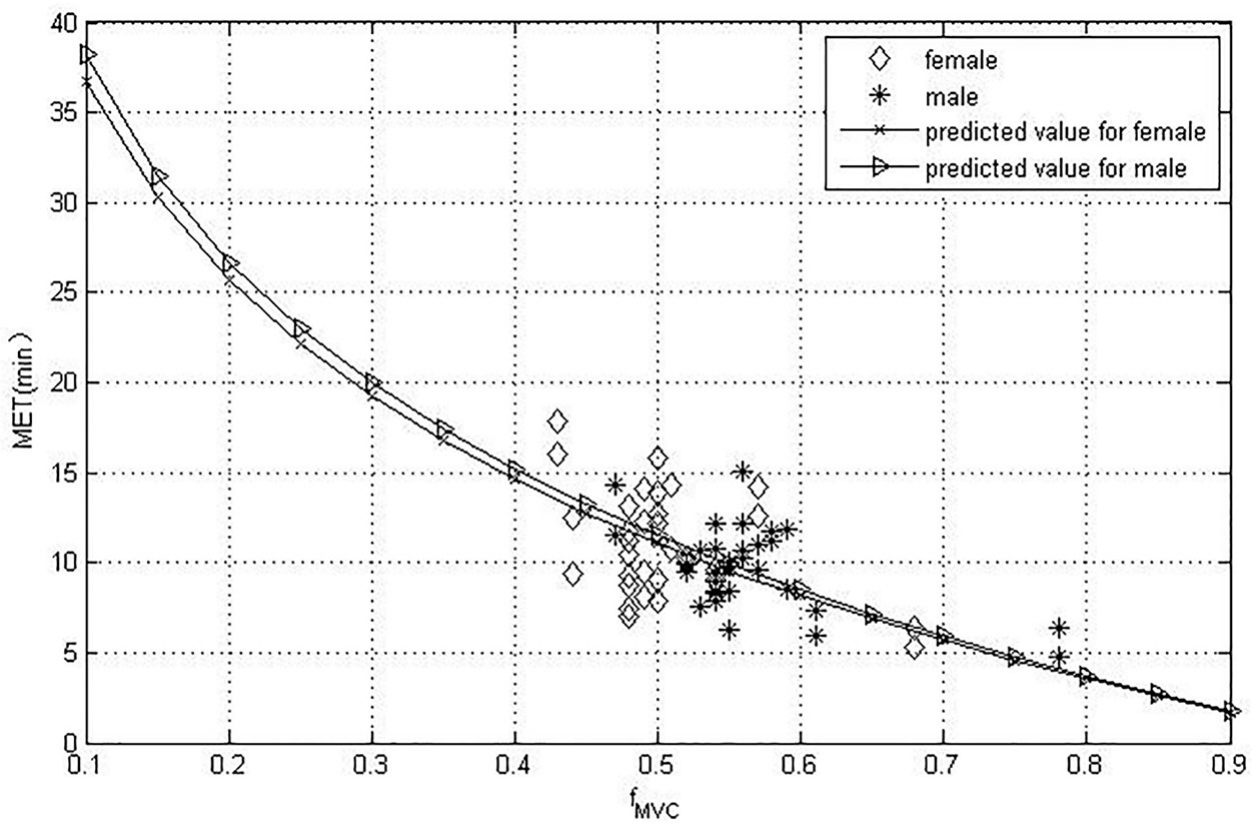
Predicted MET and scatter plot of MET versus f_*MVC*_.

The MAD values for the participant were calculated according to Eq (7) and are shown in Table 4. Equations (9) and (11) were the best fitted models and were chosen as MET prediction models for female and male participants because they had the lowest MAD values, respectively. Fig 3 shows the predicted MET using equations (9) and (11) and the scatter plot of the MET data versus the f_*MVC*_.

## Discussions

To verify MET models, comparison between proposed models and existing models are usually adopted [26, 27, 35]. In our two-handed pulling task, the participants terminated their trials mainly due to pains on feet/shanks and hands in their two-handed pulling tasks based on the results of the CR-10 (Table 2). In the static one-handed pallet truck pulling task [27], however, the participants stopped their pulling task mainly due to pains on hand/wrist and elbow. All the hand models and elbow models in the literature [20, 28–32] underestimated the MET for one-handed pulling task in a previous study [27]. The predicted MET estimated by the joint-based models [36] and back/hip models [28, 30] provided better fit than those of the general and upper limb models in the one-handed pulls in the literature [27]. Normally, two-handed pulling is adopted when the workers feel hard to pull using one hand. People could pull more easily at the same load and sustain a longer time when pulling with two hands. The hand and elbow models [20, 28–32] may not fit the two-handed pulling tasks since they were established using one-handed pulling data.

To verify our MET prediction models, the back/hip models [28, 30], joint-specific models [36], force-muscle model [14] and the one-handed models [27] were adopted for comparison purposes as shown in Table 5. All the models [27, 28, 30, 36] underestimated the MET of our two-handed pulling tasks with MAD range of 3.17–9.63 min for females and 2.91–8.59 min for males, respectively. Although the posture 5 in Rohmert’s back/hip model [28] was quite different from that of ours, the predicted MET based on this posture fitted our two-handed pulling tasks better than all the others. This might be attributed to the similarity of muscle groups recruited between Rohmert’s posture 5 [28] and that of our two-handed pulling tasks. Both these two postures mainly employed muscles on the back/hip and lower limbs.

**Table 5:** MAD (min) using the MET models in the literature.

		Females		Males	
Models		Predicted MET	MAD	Predicted MET	MAD
Manenica [30]	Body pull	3.06 (0.65)	7.29 (2.76)	2.65 (0.56)	6.99 (2.07)
	Back muscle	2.53 (0.62)	7.82 (2.77)	2.14 (0.51)	7.50 (2.08)
Frey Law et al.[36]	Ankle	2.20 (0.44)	8.14 (2.82)	1.93 (0.35)	7.71 (2.14)
	Trunk	1.66 (0.37)	8.69 (2.84)	1.43 (0.29)	8.21 (2.17)
Yi et al. [27]	Exponential-based	4.60 (1.02)	5.75 (2.70)	3.97 (0.87)	5.67 (1.99)
	Power-based	4.21 (0.91)	6.14 (2.71)	3.65 (0.71)	5.99 (2.03)
Rohmert et al. [28]	Posture 3	1.89 (0.51)	8.46 (2.80)	1.56 (0.38)	8.08 (2.14)
	Posture 4	2.84 (0.37)	7.51 (2.84)	2.64 (0.32)	7.00 (2.15)
	Posture 5	7.28 (0.91)	3.17 (2.55)	6.78 (0.79)	2.91 (1.94)
Ma et al. [14]	*k* = 1	1.39 (0.31)	9.63 (2.86)	1.05 (0.26)	8.59 (2.18)
	k*	9.79 (2.92)	2.22 (1.69)	10.00 (2.66)	2.07 (1.53)

Note: Values within brackets are standard deviations. Measured MET for female and male participants were 11.02 min (SD = 2.98) and 9.64 min (SD = 2.31), respectively.

*k was 0.125 and 0.115 for female and male participants, respectively.

In Ma’s model [14, 37], *k* was defined as fatigue rate. This parameter was found to be 0.93 and 0.66 for male and female participants respectively for pushing tasks [35], 1.02 for drilling tasks [37] and 0.29 for one-handed pallet truck pulling tasks [27]. In our study, the predicted MET of 1.39 min (SD = 0.31) and 1.05 min (SD = 0.26) for female and male participants, respectively, were obtained if *k* was equal to 1. These predictions were far less than the actual MET of 11.02 min (SD = 2.98) and 9.64 min (SD = 2.31) for females and males, respectively. This implied that *k* in the two-handed pulling task should be lower than 1. Actually, since it was easier to pull with two hands than one hand, fatigue rate *k* for two-handed pulls would be lower than that of singe-handed ones. By calculating average predicted MET and MAD, we found best fitted MET could be obtained when assigning *k* = 0.125, 0.115 for females and males participants. The corresponding MAD was 2.22 min (SD = 1.69) and 2.07 min (SD = 1.53), respectively. Although MET under f_*MVC*_ between females and males were significantly (*p*<0.05) different in our pulling task, *k* was approximately the same for the two genders. Therefore, *k* might be task dependent.

In a previous study [27], the participants reported higher fatigue on hand/wrist and elbow, followed by low back, and leg/ankle in the one-handed pulling tasks. In our pulling tasks, however, the CR-10s of body segment fatigue were quite different from those of the previous study. The front foot/shank had the highest CR-10 scores in the current study, followed by hands and other body segments. This might be attributed to the differences of the burdens on the body segments. The external loads in the current study (40 kg & 50 kg) were higher than those in the previous study (30 kg & 40 kg). The external loads were handled using two hands in the current study but was handled using one hand in the previous one. The burdens on the upper extremities of the participants in our study were, therefore, lower than those in the previous study. The burdens on the leg in this study were, on the other hand, higher than those in the previous one as the front leg needed to resist higher ground force for balance purposes as compared to the one-handed pulling in the previous study.

Posture was insignificant to the MET. This contradicted to our hypothesis. For the subjective muscular fatigue, the CR-10 on feet/shanks ranked the highest, followed by hands. This implied that the participants terminated their pulling tasks mainly due to the fatigue on their feet/shanks. Muscular fatigue on feet/shanks, therefore, became dominant in determining the endurance time for the two-handed pulling tasks.

The participants were requested to maintain the same posture while pulling the best they could. They, however, might adjusted their postures involuntarily especially when their muscle strength were becoming weak before the end of the trial. The literature [20] has found that alternating recruitment of muscle groups was the most favorable strategy in prolong muscular exertions. Dieekn et al. [38] also indicated that their participants tended to change motor control strategy during prolong exercise. Both the alternation of muscle group recruitment and change of motor control strategy could result in posture change more or less. This could explain some of the variation in the MET.

In our simulated pulling experiment, walking was not considered due to the limitations of the space and technical difficult in data collection in the laboratory. When pulling using two hands, human gait involves cyclic movements on the lower extremities. Description of such a gait may require the measure of gait parameters such as cadence, stride length, and so on. Human gait could have cyclic effects on pulling force exertion. Such effects were, however, not considered in the current study and may be research topics in the future.

## Conclusions

A simulated two-handed pulling experiment was performed. We found that posture significantly affected MVC_before_ and MVC_after_ but was insignificant to MET. Feet/shanks were the body segments most likely to suffer muscular fatigue for the two-handed pulling, followed by hands and other body segments. MET models were obtained for females and males, respectively. The MET models are beneficial for job design and work-rest scheduling for workers where static two-handed pulling tasks are commonly performed.

## Supporting information

S1 Raw Data (XLSX)
