# Correction: Pulling strength, muscular fatigue, and prediction of maximum endurance time for simulated pulling tasks

**DOI:** 10.1371/journal.pone.0209882

**Published:** 2018-12-20

**Authors:** Cannan Yi, Kai Way Li, Fan Tang, Huali Zuo, Liang Ma, Hong Hu

In the Funding section, the grant number from the National Science Foundation, China is listed incorrectly. The correct grant number is: 71801089.
